# Comparison of Sedation With Ketamine-Propofol Versus Propofol-Fentanyl for Elderly Patients Undergoing Prostate Biopsy: A Retrospective Observational Study

**DOI:** 10.7759/cureus.42650

**Published:** 2023-07-29

**Authors:** Kentaro Fukano, Yusuke Iizuka, Takahiro Ueda, Yu Fukuda, Yuji Otsuka, Masamitsu Sanui

**Affiliations:** 1 Department of Anesthesiology and Critical Care, Jichi Medical University Saitama Medical Center, Saitama, JPN

**Keywords:** deep sedation, elderly, fentanyl, propofol, ketamine, procedural sedation

## Abstract

Background: Procedural sedation is increasingly used for elderly patients, but there is no established ideal method for elderly patients who are prone to respiratory and circulatory depression. This study aims to investigate the association of respiratory complications and the combination of ketamine-propofol versus fentanyl-propofol in elderly patients undergoing prostate biopsy requiring deep sedation.

Methods: This was a single-center, retrospective, observational study conducted from April 2020 to March 2021. We included male patients aged 65 years and older scheduled for prostate biopsy under procedural sedation. Ketamine-propofol and fentanyl-propofol were administered at the discretion of the anesthesiologist. The primary outcome was the need for assisted ventilation. The secondary outcome was the duration of oxygen saturation (SpO_2_) below 90%.

Results: We enrolled 120 patients over 65 years, and 92 patients were included in the final analysis. The anesthesiologist administered an initial dose of ketamine and propofol of 1:1 to 1:4 of 1.0 mg kg^-1^ (interquartile range: 0.98 to 1.17) or administered an initial dose of fentanyl of 0.05 to 0.1 mg and a target-controlled infusion of propofol of 2.8 μg ml^-1^ (interquartile range: 2.0 to 3.0) followed by additional doses at the discretion of the anesthesiologist. Ketamine-propofol was associated with a reduced need for assisted ventilation and a shorter duration of SpO2 below 90% than propofol-fentanyl (95.7% vs. 4.3%, P < 0.05; 0.64 minutes vs. 0.17 minutes, P = 0.26).

Conclusions: Ketamine-propofol is associated with a significantly reduced need for assisted ventilation compared to propofol-fentanyl during procedural sedation and analgesia for procedures requiring deep sedation for the elderly.

## Introduction

For painful or difficult procedures, procedural sedation and analgesia (PSA) allow performing the procedure while administering sedatives and analgesics to relieve the patient's pain and anxiety [1]. It was reported that respiratory complications such as hypoxia (9.9%) and apnea (7.2%) were most common during PSA in Japan, and these complications did not lead to serious adverse events. However, accidents such as hypoxic encephalopathy under sedation have in fact been reported and continue to occur [2].

The National Institutes of Health reported that "some of the already old countries in 2020 will continue to see rapid growth of the population 65 and older, with projected increases of higher than 10 percentage points. Examples include Japan (percent 65 and older in 2020 vs. 2050: 29.2, 40.1)" [3]. Inevitably, procedural sedation is increasingly performed for elderly patients, but there is no ideal method of sedation defined for elderly patients prone to respiratory and circulatory depression [4,5].

A combination of propofol and fentanyl is used when a patient needs deep sedation during a painful procedure. Deep sedation results from the use of propofol, and fentanyl is a commonly used opioid in PSA. However, when deep sedation is targeted, this combination is likely to cause respiratory depression, especially for elderly patients. The combination of ketamine and propofol is also used for PSA, and there are several systematic reviews showing that this combination causes fewer respiratory complications than propofol alone [6-9]. This suggests that the combination of ketamine and propofol is more suitable for the elderly and patients who are deeply sedated receiving PSA. However, no studies have examined the effects of the combination of ketamine and propofol during procedures requiring deep sedation, such as prostate biopsy in the elderly. Our hypothesis was that the combination of ketamine and propofol is significantly less likely to cause respiratory depression than the combination of propofol and fentanyl for the elderly who are deeply sedated receiving PSA. We aimed to compare the incidence of rescue maneuvers such as assisted ventilation. We also aimed to compare the duration of oxygen saturation (SpO2) below 90%.

## Materials and methods

Study design

This single-center, retrospective, observational study was approved by the Institutional Review Board. Due to the retrospective nature of the study, the requirement for written informed consent was waived. The results of this study were reported according to the Strengthening the Reporting of Observational Studies in Epidemiology (STROBE) guidelines.

Study setting

This study was conducted at a single center in Japan. This is an urban hospital with an anesthesiology residency training program. Eighteen anesthesiologists participated in this study. Clinical data were obtained from April 2020 to March 2021.

Selection of participants

All elderly patients (≥65 years of age) undergoing prostate biopsy and sedated with propofol-fentanyl or ketamine-propofol were considered eligible for inclusion. Patients undergoing other procedures at the same time (e.g., transurethral resection of the bladder), receiving spinal anesthesia, undergoing PSA with anesthetics other than propofol-fentanyl or ketamine-propofol, and patients with missing data were excluded from this study.

Patient preparation

The prostate biopsy was performed in the operating room by a urologist. PSA is typically administered by anesthesiologists or postgraduate year 1 and 2 residents who are on the anesthesia rotation under the direct supervision of an anesthesiologist. Prophylactic antimicrobial agents were administered orally with levofloxacin before the procedure, and no other prophylactic antimicrobial agents were administered before the procedure. The patient walked into the operating room, lay down on the operating table, received local anesthesia with 0.5% or 1% xylocaine subcutaneously, and an intravenous catheter was inserted. The patient was given intravenous fluid. The patient was fitted with electrocardiography (ECG) and SpO2 monitors, and blood pressure was automatically measured every 2.5 minutes. The patient received supplemental oxygen of 6 L min^-1^ (6.0 to 6.0), administered through a transparent face mask connected to an anesthesia machine, and a capnometer was connected to the face mask.

The medications and their administration for PSA were left to the discretion of the anesthesiologist. Dosing of sedatives and analgesics was also left to the discretion of the anesthesiologist. Each anesthesiologist used both methods in PSA. In the propofol-fentanyl group, fentanyl was given, followed by a target-controlled infusion of propofol (Diprifusor^TM^, Terumo, Tokyo, Japan). In the ketamine-propofol group, medications were administered in one of the three following patterns: Group I received a continuous infusion of propofol and ketamine, Group II received a continuous infusion of propofol and an intermittent bolus of ketamine, and Group ­III received intermittent bolus infusion of propofol and ketamine. Once the patient was deeply sedated (no response after prodding or shaking), the patient was placed in the open leg position and the procedure was undertaken by the urologist. After the procedure, at the anesthesiologist’s discretion, with normalization of consciousness (patients can respond to name calls and follow instructions), respiration, and circulation, the patient was transported out of the operating room.

Data collection and definition

Clinical data were collected retrospectively from anesthesia records and medical records. Information, such as the patient's height, weight, body mass index (BMI), comorbidities, American Society of Anesthesiologists (ASA) classification, and airway assessment, was collected from the medical records. From the anesthesia record, the following information was obtained: vital signs (blood pressure, heart rate, respiratory rate, SpO2, and end-tidal carbon dioxide (CO2)), choice of sedatives, the total dose of sedatives, choice of analgesics, the total dose of analgesics, intervention for respiratory complications (assisted ventilation because of prolonged apnea, placement of a supraglottic airway), procedure time, anesthesia time, induction time (the time from the start of induction of anesthetic agent to the start of surgery), and recovery time (the time from the end of surgery to the end of anesthesia in the operating room).

Measurements

The primary outcome was the need for assisted ventilation because of prolonged apnea. The secondary outcomes were the duration of SpO2 below 90%, the need for intubation, the total amount of fluid infused, use of vasopressors, procedure time, anesthesia time, induction time (the time from the start of anesthesia to the start of surgery), recovery time (the time from the end of surgery to the end of anesthesia), prostate volume, and the frequency of cancer detection and biopsy. Assisted ventilation, intubation, and the use of vasopressors were used at the discretion of the anesthesiologist.

Statistical analysis

Simple descriptive statistics with 95% confidence intervals (95% CIs) were used to analyze the data. Continuous variables are expressed as means (standard deviation, SD) or medians (interquartile range, IQR). Categorical variables were presented as frequencies and percentages. Continuous variables were compared using Student’s t-test or the Wilcoxon rank-sum test depending on the distribution. Categorical variables were compared with the chi-square test when appropriate; otherwise, Fisher’s exact test was used. Multivariable logistic regression analysis was performed and adjusted for confounding factors, to determine the association of propofol plus ketamine and associated ventilation. Age, BMI, ASA class (2 or greater), and analgesic use (ketamine or fentanyl) were used as explanatory variables to be used in the logistic regression analysis [10-12]. R Core Team (2021) software (R Foundation for Statistical Computing, Vienna, Austria) was used for analysis and preparing graphs. All P-values were two-tailed, with P < 0.05 considered statistically significant. Sensitivity analysis was performed for patients aged ≥ 75 years.

## Results

**Figure 1:**
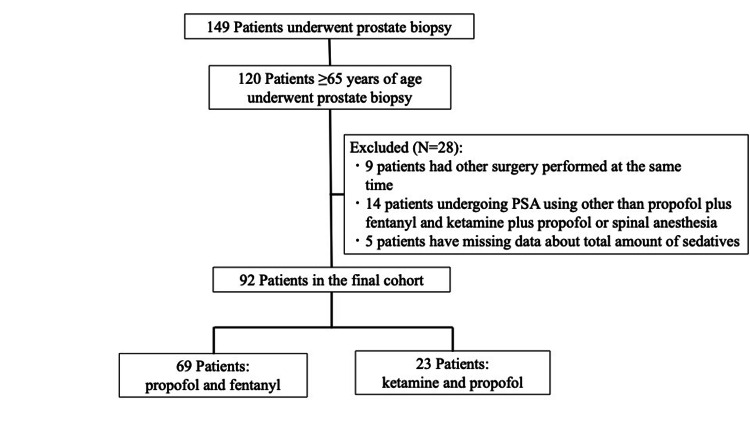
Patient flow PSA: procedural sedation and analgesia.

Of 149 patients undergoing prostate biopsy from April 2020 to March 2021, 120 were over 65 years of age. Nine patients who underwent other procedures at the same time, 14 patients who underwent PSA with other than propofol-fentanyl or propofol-ketamine or spinal anesthesia, and five patients with missing data were excluded. Ninety-two patients were included in the final analysis with 23 patients in the ketamine-propofol group and 69 patients in the propofol-fentanyl group (Figure 1).

Baseline patient characteristics are shown in Table 1. ASA classification, age, and BMI were well-balanced in both groups. There were more patients with chronic obstructive pulmonary disease (COPD) in the ketamine-propofol group (26.1% vs. 4.3%, P < 0.05). Anesthesiologists administered an initial dose of ketamine and propofol of 1:1 to 1:4 of 1.0 mg kg^-1^ (IQR: 0.98 to 1.17) in the ketamine-propofol group (if an initial dose of ketamine and propofol was 1.0 mg kg^-1^ in a 1:4 ratio, we administered ketamine at 0.2 mg/kg and propofol at 0.8 mg/kg separately) and administered an initial dose of fentanyl of 0.05 to 0.1 mg and a target-controlled infusion of propofol of 2.8 μg ml^-1^ (IQR: 2.0 to 3.0) in the propofol-fentanyl group followed by additional doses as needed. The total amount of propofol in the propofol-fentanyl group was more than that in the ketamine-propofol group (230 mg vs. 158 mg, P < 0.05). The incidence of comorbidities, including hypertension, diabetes mellitus, ischemic heart disease, and obstructive sleep apnea syndrome (OSAS), was similar in the two groups.

**Table 1:** Patient characteristics IQR: interquartile range; BMI: body mass index; ASA: American Society of Anesthesiologists: IHD: ischemic heart disease; COPD: chronic obstructive pulmonary disease; OSAS: obstructive sleep apnea syndrome.

	Propofol-fentanyl group (N = 69)	Ketamine-propofol group (N = 23)	P-value
Age, years, IQR	73 (70 to 77)	73 (68 to 78)	0.59
BMI, kg/m^2^, IQR	24.2 (22.0 to 26.0)	23.4 (22.3 to 24.7)	0.46
ASA, n (%)			
Class I	6 (8.7)	2 (8.7)	0.98
Class II	55 (79.7)	18 (78.3)	
Class III	8 (11.6)	3 (13.0)	
Hypertension, n (%)	35 (50.7)	14 (60.9)	0.55
Diabetes mellitus, n (%)	17 (24.6)	6 (26.1)	1.00
IHD, n (%)	10 (14.5)	3 (13.0)	1.00
Atrial fibrillation, n (%)	3 (4.3)	2 (8.7)	0.79
COPD, n (%)	3 (4.3)	6 (26.1)	0.01
OSAS, n (%)	27 (39.1)	9 (39.1)	1.00
Total amount of propofol, mg, IQR	230 (200 to 250)	158 (125 to 200)	<0.001
Total amount of analgesics, mg, IQR	0.1 (0.1 to 0.15)	60 (50 to 99]	<0.001

Table 2 shows the results for the primary and secondary outcomes. Ketamine-propofol was associated with a lower incidence of requiring assisted ventilation (95.7% vs. 4.3%, P < 0.05). Ketamine-propofol was associated with a shorter duration of SpO2 below 90% but the difference was not statistically significant (0.64 minutes vs. 0.17 minutes, P = 0.26). Although the frequency of biopsy was almost the same, the procedure duration and anesthesia time in the ketamine-propofol group was longer than in the propofol-fentanyl group. The rate of detection of cancer in the ketamine-propofol group was higher than in the propofol-fentanyl group.

**Table 2:** Primary and secondary outcomes SD: standard deviation; IQR: interquartile range; SpO2: oxygen saturation.

	Propofol-fentanyl group (N = 69)	Ketamine-propofol group (N = 23)	P-value
Primary outcome			
Assisted ventilation needed, n (%)	66 (95.7)	1 (4.3)	<0.001
Secondary outcomes			
Duration of SpO2 < 90%, minutes, SD	0.64 (1.91)	0.17 (0.58)	0.256
Intubation, n (%)	2 (2.9)	0 (0.0)	1.00
Total amount of infusion, ml, IQR	250 (200 to 350)	400 (300 to 438)	<0.001
Vasopressor, n (%)	21 (30.4)	2 (8.7)	0.07
Prostate volume, ml, IQR	27.8 (19.8 to 38.4)	20.0 (15.0 to 29.6)	0.06
Frequency of biopsy, times, IQR	16 (16 to 18)	16 (16 to 16)	0.22
Cancer detection, n (%)	49 (71.0)	19 (82.6)	0.41
Procedure time, minutes, IQR	12 (10 to 15)	15 (12 to 19)	0.06
Anesthesia time, minutes, IQR	33 (28 to 36)	35 (33 to 41)	0.02
Induction time, minutes, IQR	12 (10 to 14)	13 (11 to 17)	0.15
Recovery time, minutes, IQR	7 (6 to 10)	9 (7 to 10)	0.75

Table 3 shows the method used for the administration of ketamine-propofol. Continuous infusion of propofol and intermittent infusion of ketamine was most frequently performed. Only one patient was ventilated in the group receiving a continuous infusion of propofol and a continuous infusion of ketamine. The amount of propofol and ketamine was least in the group treated with a continuous infusion of propofol and intermittent infusion of ketamine.

**Table 3:** Primary and secondary outcomes in ketamine and propofol groups IQR: interquartile range; Group I: propofol continuous infusion + ketamine continuous infusion; Group II: propofol continuous infusion + ketamine intermittent infusion; Group III: propofol intermittent infusion + ketamine intermittent infusion.

	Group I (N = 6)	Group II (N = 13)	Group III (N = 4)	P-value
Total amount of propofol, mg, IQR	186 (139 to 267)	157 (119 to 189)	175 (143 to 200)	0.43
Total amount of ketamine, mg, IQR	100 (99 to 104)	50 (40 to 60)	75 (58 to 93)	0.03
Assisted ventilation, n (%)	1 (16.7)	0 (0.0)	0 (0.0)	0.23
Procedure time, minutes, IQR	8.5 (7.3 to 15.0)	15.0 (12.0 to 18.0)	18.5 (16.5 to 20.0)	0.15
Anesthesia time, minutes, IQR	34.5 (33.3 to 35.0)	39.0 (35.0 to 44.0)	32.0 (27.3 to 36.3)	0.18
Induction time, minutes, IQR	13.0 (11.5 to 16.0)	16.0 (12.0 to 18.0)	9.0 (7.8 to 10.5)	0.04
Recovery time, minutes, IQR	9.5 (9.0 to 10.8)	9.0 (7.0 to 10.0)	4.5 (1.5 to 7.3)	0.08

Table 4 shows the results of the logistic regression analysis. Ketamine was associated with a significantly lower incidence of the need for assisted ventilation compared to fentanyl (odds ratio: 0.0009, 95% CI: 0.000018 to 0.01, P < 0.05).

**Table 4:** Logistic regression analysis ASA: American Society of Anesthesiologists.

Explanatory variables	Odds ratio	95% confidence interval	P-value
Age	1.17	0.93 to 1.53	0.197
Ketamine versus fentanyl	0.0009	0.000018 to 0.01	<0.05
ASA class 2 or 3 (versus 1)	0.49	0.004 to 21.2	0.767
Body mass index	1.37	0.91 to 2.18	0.151

The sensitivity analysis for patients aged 75 years and older gave similar results. Ketamine-propofol was associated with a lower incidence of requiring intervention with assisted ventilation than propofol-fentanyl (100.0% vs. 12.5%, P < 0.05).

## Discussion

To the best of our knowledge, this is the first study to examine the effects of the combination of ketamine and propofol on elderly patients undergoing procedures requiring deep sedation. One study examining the effects of the combination of ketamine and propofol on the elderly was conducted on elderly patients undergoing endoscopic retrograde cholangiopancreatography (ERCP), but the targeted sedation depth was moderate to deep [13]. Although the depth of sedation was not objectively evaluated in our study, we thought that the sedation depth was deep because anesthesiologists could keep the patient immobile during prostate biopsies. In the present study, ketamine and propofol were associated with a lower incidence of the need for mask ventilation (95.7% vs. 4.3%, P < 0.05). The present study suggests that the combination of ketamine and propofol may be safely used in the elderly for procedures that require deep sedation, such as prostate biopsy.

To discuss administering deep sedation to elderly patients with the combination of ketamine and propofol, there are three points to consider: respiratory complications, hemodynamic complications, and delay in discharge time. First, it was unclear whether the combination of ketamine and propofol during PSA significantly reduces respiratory complications in elderly patients who are likely to develop respiratory complications. However, systematic reviews have concluded that the combination of ketamine and propofol causes significantly less respiratory depression than propofol alone [6-9]. Analgesics such as fentanyl should be used during painful procedures such as prostate biopsy, and it is likely to cause respiratory complications when the combination of propofol-fentanyl is used. In a study comparing four groups of propofol-based sedation in the elderly including no analgesics, a single dose of ketamine (0.4 mg/kg), a single dose of sufentanil (0.1 μg/kg), and a single dose of dexmedetomidine (0.4 μg/kg), the incidence of hypoxia was significantly lower in the ketamine group than in the sufentanil group [14]. In the present study, there was no difference in ASA classifications in the two groups, and patients in the ketamine-propofol group had more underlying diseases (COPD) that may predispose them to respiratory complications. There were significantly fewer interventions for respiratory complications in the ketamine-propofol group and two patients were intubated with a supraglottic airway due to prolonged apnea and difficult ventilation in the propofol-fentanyl group. This may be because the dissociative effect of ketamine caused a reduction of the dosage of propofol until the target depth of sedation was reached, and ketamine itself preserved spontaneous breathing compared to fentanyl. These results support the hypothesis of the present study and are consistent with previous studies.

Second, a systematic review concluded that the combination of ketamine and propofol is less likely to cause hypotension and is associated with greater hemodynamic stability than propofol alone [7]. In elderly patients who easily become hypotensive with the administration of sedatives and analgesics, the use of ketamine and propofol is likely to be advantageous. The results of the present study suggest that the combination of ketamine and propofol may lead to greater hemodynamic stability due to a lesser need for vasopressor treatment. However, since the amount of infusion was significantly higher in the ketamine-propofol group, we do not know whether the hemodynamic stability was due to infusion volume or the combination of ketamine-propofol. The results of the present study do not allow us to conclude whether the use of ketamine-propofol stabilizes the hemodynamics during PSA in the elderly.

Third, it was reported that the combination of ketamine-propofol led to a delay in discharge time compared to propofol alone [15]. The use of sedatives and analgesics during PSA in the elderly is more likely to cause delayed arousal than in healthy young people. In the present study, there was no delay in recovery time from anesthesia between the propofol-fentanyl group and the ketamine-propofol group. The procedure and anesthesia time were longer in the ketamine-propofol group than in the propofol-fentanyl group. We surmise that this was due to the skill of the urologists or involuntary movements and emergence reactions in patients treated with ketamine.

Limitations

Due to the retrospective nature of this observational study, biases may be present. There is a selection bias because it is up to the discretion of the anesthesiologist to decide which medications were used. Each anesthesiologist performed both methods of PSA. However, it is possible that patients at high risk for respiratory complications may have PSA with ketamine-propofol. Therefore, there were more COPD patients in the ketamine-propofol group than in the propofol-fentanyl group. Conversely, it is possible that we performed PSA for those who were not difficult to ventilate under a depth of sedation similar to that of general anesthesia with the propofol-fentanyl combination. Since the elderly are included as a factor for difficulty with mask ventilation, we do not think that PSA in the elderly was likely to be performed on the assumption of mask ventilation. In this study, the number of prostate biopsy punctures, procedure time, and prostate cancer detection rate were evaluated as evidence that the prostate biopsy procedure was performed safely and accurately, with no significant differences between the two groups. If we need to perform PSA under more than deep sedation, it may be possible to say that the combination of ketamine and propofol may be more likely to reduce respiratory complications than the combination of propofol and fentanyl. To partially account for this limitation in study design, logistic regression analysis was performed on confounders reported in previous studies to be associated with assisted ventilation to adjust for confounders as much as possible.

The depth of sedation was not objectively evaluated in this study. Immobilization of the patient causes an enhanced detection rate of cancer [16]. Therefore, it has been reported that PSA for prostate biopsy requires deep sedation [17]. Sedation depth may differ with different anesthesiologists. Each anesthesiologist used both methods of PSA. The goal of performing prostate biopsies under deep sedation sufficient to perform it safely and accurately could be achieved despite the various drug administrations at the discretion of anesthesiologists. Surgeon satisfaction is related to induction time and safe and accurate performance of prostate biopsies safely due to patients' immobilization. Induction time was not significantly different between the two groups. Although sedation depth is an indicator of whether patients were immobilized, we did not objectively evaluate sedation depth. However, prostate biopsy was performed safely and accurately in terms of the number of prostate biopsy punctures, procedure time, and prostate cancer detection rate. We assumed that surgeons could be satisfied with sedation depth because patients were immobile during prostate biopsy. The procedure was considered to be performed under deep sedation because the procedure time in this study was not significantly longer than that in previous studies and a higher incidence of positive pressure ventilation occurred than under deep sedation in previous studies [18,19]. Considering the cancer detection rate and procedure time in both groups, the sedation depth was not significantly different between the two groups.

There was variation in the dosages of ketamine and propofol administered. The optimal dosage and combination ratio of ketamine and propofol is not known. There are some studies on children, but no studies on the elderly [20-22]. In this study, the more common ratio of propofol and ketamine was 2:1 or 3:1 and the most common method of administration was continuous infusion of propofol and intermittent infusion of ketamine. It is a commonly used method based on previous studies. Further study on the doses used is needed.

## Conclusions

The combination of ketamine and propofol is associated with a significantly lower incidence of needing assisted ventilation compared to patients treated with propofol-fentanyl during PSA for procedures requiring deep sedation in the elderly. Randomized controlled multicenter trials are needed to confirm the findings of the present study. Further studies are also needed to determine the optimal dose and ratio of medications used.
